# Genome-wide association study of leaf rust resistance in Russian spring wheat varieties

**DOI:** 10.1186/s12870-020-02333-3

**Published:** 2020-10-14

**Authors:** Irina N. Leonova, Ekaterina S. Skolotneva, Elena A. Salina

**Affiliations:** 1 The Federal Research Center Institute of Cytology and Genetics of Siberian Branch of the Russian Academy of Sciences, Novosibirsk, 630090 Russia; 2 Kurchatov Genomics Center Institute of Cytology and Genetics SB RAS, Novosibirsk, 630090 Russia

**Keywords:** Common wheat, Leaf rust, *Puccinia triticina*, GWAS, *Lr* genes

## Abstract

**Background:**

Leaf rust (*Puccinia triticina* Eriks.) is one of the most dangerous diseases of common wheat worldwide. Three approaches: genome-wide association study (GWAS), marker-assisted selection (MAS) and phytopathological evaluation in field, were used for assessment of the genetic diversity of Russian spring wheat varieties on leaf rust resistance loci and for identification of associated molecular markers.

**Results:**

The collection, consisting of 100 Russian varieties of spring wheat, was evaluated over three seasons for resistance to the native population of leaf rust specific to the West Siberian region of Russia. The results indicated that most cultivars showed high susceptibility to *P. triticina,* with severity ratings (SR) of 60S–90S, however some cultivars showed a high level of leaf rust resistance (SR < 20MR-R). Based on the results of genome-wide association studies (GWAS) performed using the wheat 15 K genotyping array, 20 SNPs located on chromosomes 6D, 6A, 6B, 5A, 1B, 2A, 2B and 7A were revealed to be associated with leaf rust resistance. Genotyping with markers developed for known leaf rust resistance genes showed that most of the varieties contain genes *Lr1*, *Lr3a*, *Lr9, Lr10*, *Lr17a*, *Lr20, Lr26* and *Lr34*, which are not currently effective against the pathogen. In the genome of three wheat varieties, gene *Lr6Ai = 2* inherited from *Th. intermedium* was detected, which provides complete protection against the rust pathogen. It has been suggested that the QTL mapped to the chromosome 5AS of wheat cultivar Tulaikovskaya-zolotistaya, Tulaikovskaya-10, Samsar, and Volgouralskaya may be a new, previously undescribed locus conferring resistance to leaf rust. Obtained results also indicate that chromosome 1BL of the varieties Sonata, Otrada-Sibiri, Tertsiya, Omskaya-23, Tulaikovskaya-1, Obskaya-14, and Sirena may contain an unknown locus that provides a resistance response to local population.

**Conclusions:**

This study provides new insights into the genetic basis of resistance to leaf rust in Russian spring wheat varieties. The SNPs significantly associated with leaf rust resistance can be used for the development and application of diagnostic markers in marker-assisted selection schemes.

## Background

Bread wheat (*T. aestivum* L.) is one of the most valuable food and feed crops and one of the main sources of dietary protein and carbohydrates. The Russian Federation, along with China, India and the United States, is one of the world’s largest producers and exporters of bread wheat. The main characteristics of modern varieties include high grain yield and quality, adaptability to environmental factors and resistance to diseases and insects.

Among the diseases causing significant yield losses in bread wheat, leaf rust (*Puccinia triticina* Eriks.) is the most widespread and dangerous one. In Russia, this disease occurs annually in all regions of winter and spring wheat cultivation, and accounts for crop losses of 40% or more [1, 2]. Significant damage from leaf rust occurs in the Volga, Central, North Caucasus and Ural regions. According to long-term studies, the severity of leaf rust in Western Siberia has also increased drastically over the past 20 years [3]. Since the early 2000s, the epiphytoties of leaf rust were observed in 2000, 2001, 2005, 2007, 2008, 2010 and 2011. The main factors, in addition to weather conditions, contributing to this process, include the changed virulence and aggressiveness of the pathogen population, combined with the high susceptibility of wheat varieties. Consequently, in recent years much attention has been paid to the creation of varieties with a genetic basis for leaf rust resistance, to prevent crop losses and reduce the pesticide load on the environment.

To date, more than 100 *Lr* (leaf rust) genes with permanent and temporary symbols controlling seedling and adult plant resistance to leaf rust have been described in wheat. Of these, more than half were inherited from the genomes of wild and cultivated wheat relatives: *Secale cereale*, *Ae. tauschii*, *Ae. speltoides*, *T. timopheevii*, *Th. elongatum*, *Th. intermedium*, *T. dicoccoides*, *Ae. ventricosa*, etc. [4]. Different approaches are used to postulate the presence of *Lr* genes in wheat cultivars and breeding lines. The conventional method includes phytopathological screening in response to inoculation of plants by pathogen isolates, with further comparison to the resistance/susceptibility reactions of a set of isogenic lines and wheat cultivars containing known *Lr* genes. This method is used to infer the presence in the genome of single genes and previously unknown resistance factors [5–7].

Another, more modern approach involves the application of DNA markers (SSRs, STS, SCAR, CAPS) developed for known leaf rust resistance genes [8, 9]. However, despite the fact that molecular markers are available for almost all *Lr* genes, only a small number of them are diagnostic and recommended for use in marker-assisted selection (http://maswheat.usdavis.edu/protocols). Most of the markers were developed using mapping populations derived from bi-parental crosses, which is one of the reasons for the decrease or complete absence of their specificity in the detection of *Lr* genes in another genetic background [10–12].

The most modern methodological approach, based on the application of a large number of SNP markers and the principles of linkage disequilibrium (LD), is the genome wide association study (GWAS). Currently, GWAS is widely used to identify the genetic factors that determine various agronomically important characteristics of agricultural plants, including resistance to fungal diseases, the duration of the vegetative period, grain yield and quality [13–16]. GWAS allows us to determine the presence of both known resistance genes and previously unidentified loci [17, 18]. For example, using this approach, a collection of 338 cultivated varieties of spring soft wheat was studied, and 46 QTLs responsible for resistance to leaf rust at seedling and adult plant stages were detected [19]. In another study, potentially new *Lr* loci were identified in the genome of spring bread wheat using a panel consisting of 1032 varieties [20].

Data on the genetic resistance of varieties created in the USSR and varieties of modern selection are practically absent, and what little exists is not systematized and is often only weakly indicative. There are few examples of published results, which suggest that released varieties from selections of the USSR contain ineffective genes *Lr3*, *Lr10*, *Lr13* [21–24]. Based on the results of molecular marker analysis of modern Russian varieties from the Volga region and Krasnodar breeding, it was assumed that they probably contain genes *Lr9*, *Lr19*, *Lr26,* as well as unknown genes from *Agropyron* ssp. [25, 26].

Information on the level of resistance of modern wheat varieties and genetic basis of resistance is needed to expand the pool of resistant cultivars available for breeding. The aim of this study was: 1) to assess the level of susceptibility of Russian spring wheat varieties to the natural population of leaf rust; and 2) to postulate the presence of *Lr* genes based on the results of GWAS and molecular marker analysis specific to known *Lr* genes.

## Results

### Field resistance of wheat varieties to leaf rust

Weather conditions in the West Siberian region in 2016–2018 favored the development of plant infection with the leaf rust pathogen (Additional file 1: Table S1). The growing season of 2018 was characterized by lower temperatures in May and significant overmoistening in May – June comparing with 2016–2017. The first symptoms of the rust infection in 2018 appeared a week later than in other years. A field evaluation of the resistance of 100 wheat varieties to the natural population of leaf rust specific to the West Siberian region showed that 38 cultivars displayed high susceptibility (IT = 4, 60–90% on the Cobb’s scale) on both experimental fields over three seasons (Table 1). The severity rating of the remaining varieties varied depending on both the year of testing and the experimental fields. ANOVA performed on the results of a three-year evaluation of the susceptibility of cultivars to the pathogen indicated significant differences (*p* < 0.001) between genotypes, environments and the experimental fields (Table 2).

**Table 1:** List of spring wheat varieties, their origin, leaf rust severity and postulated *Lr* resistance genes

Variety	Region/Originator	Cluster^a^	Infection type/severity^b^					Postulated *Lr* genes
			Field-1-2016	Field-1-2017	Field-1-2018	Field-2-2016	Field-2-2017	
Kuibyshevskaya-2	Samarskaya Oblast / Samarskii NIISKH	V	4/90S	4/90S	4/70S	4/60S	4/80S	*Lr34*
Lutescens-840		I	4/70S	4/40S	4/70S	4/60S	4/60S	*Lr10*
Samsar		IV	0/R	0/R	0/R	0/R	0/R	*Lr3a, Lr17a, Lr20, Lr6Ai = 2*
Tulaikovskaya-belozernaya		I	4/70S	4/90S	3/40MS	4/30S	4/30S	*Lr3a*
Tulaikovskaya-stepnaya		I	4/50S	4/50S	3/20MS	4/30S	4/30S	*Lr3a, Lr10, Lr17a*
Tulaikovskaya-zolotistaya		IV	0/R	0/R	0/R	0/R	0/R	*Lr3a, Lr6Ai = 2*
Tulaikovskaya-1		IV	2/15MR	0/R	2/30MR	0/R	0/R	*Lr3a, Lr17a*
Tulaikovskaya-10		IV	0/R	0R	0/R	0/R	0/R	*Lr3a, Lr20, Lr6Ai = 2*
Kinelskaya-40		IV	4/80S	4/80S	3/40MS	4/40S	4/60S	*Lr10*
Kinelskaya-60		I	2/20MR	3/20MS	2/15MR	3/10MS	3/20MS	*Lr17a, Lr26*
Volgouralskaya		IV	0/R	1/5MR	1/5MR	0/R	1/5MR	*Lr19*
Saratovskaya-29	Saratovskaya Oblast / NIISKH Yugo-Vostoka	IV	4/90S	4/90S	4/70S	4/70S	4/70S	*Lr3a, Lr10*
Saratovskaya-42		IV	4/80S	4/80S	4/80S	–	4/70S	*Lr3a*
Lutescens-62		IV	4/70S	4/80S	4/80S	–	4/70S	*Lr3a*
Lutescens-80	Altaiskii Krai / Altaiskii NIIZIS	III	4/60S	4/80S	4/80S	4/60S	4/60S	*Lr3a, Lr10*
Lutescens-85		IV	3/60MS	3/50MS	3/30MS	3/20MS	3/40MS	*Lr1, Lr3a, Lr17a*
Lutescens-148		III	4/80S	4/60S	4/60S	4/70S	4/90S	*Lr3a, Lr10, Lr20*
Altaiskii-prostor		III	4/60S	4/70S	4/70S	4/20S	4/50S	*Lr9, Lr10, Lr17a, Lr20*
Altaiskaya-92		V	4/70S	4/80S	4/80S	4/10S	4/40S	*Lr3a, Lr17a, Lr34*
Altaiskaya-99		V	3/10MS	3/30MS	3/15MS	3/20MS	3/20MS	*Lr3a, Lr9*
Altaiskaya-100		IV	3/40MS	3/60MS	3/40MS	3/10MS	3/10MS	*Lr1, Lr3a*
Altaiskaya-325		V	4/80S	4/90S	4/60S	4/70S	4/90S	*Lr3a, Lr10, Lr17a, Lr20*
Altaiskaya-530		V	4/60S	4/70S	4/70S	4/40S	4/50S	*Lr1, Lr3a*
Erythrospermum-72		II	4/60S	4/80S	3/50MS	4/70S	4/90S	*Lr1*
Sibirskaya-12	Novosibirskaya Oblast / SibNIIRS	I	4/80S	4/80S	4/50S	4/50S	4/60S	*Lr3a, Lr10*
Novosibirskaya-15		III	4/80S	4/90S	4/80S	4/60S	4/70S	*Lr1, Lr3a, Lr10*
Novosibirskaya-20		III	4/70S	4/90S	4/80S	3/30MS	3/40MS	*Lr3a, Lr10*
Novosibirskaya-22		III	4/80S	4/70S	4/60S	4/60S	4/70S	*Lr3a, Lr10*
Novosibirskaya-29		V	4/90S	4/90S	4/80S	4/70S	4/70S	*Lr3a, Lr10*
Novosibirskaya-67		V	4/90S	4/90S	4/80S	4/80S	4/90S	*Lr17a*
Novosibirskaya-81		I	4/70S	4/90S	4/70S	4/60S	4/60S	*Lr1, Lr17a, Lr20*
Novosibirskaya-89		V	4/70S	4/90S	4/60S	4/90S	4/90S	*Lr10, Lr20*
Novosibirskaya-91		IV	4/60S	4/70S	4/70S	4/60S	4/60S	*Lr1*
Lutescens-25		III	4/70S	4/90S	4/70S	4/50S	4/70S	*Lr3a, Lr10*
Obskaya-14		V	2/40MR	2/20MR	1/5MR	0/R	0/R	*Lr3a, Lr9, Lr10*
Kantegirskaya-89		V	4/60S	4/80S	4/80S	4/15S	4/15S	*Lr3a, Lr10*
Aleksandrina		V	3/25MS	3/30MS	4/50S	4/50S	4/40S	*Lr3a, Lr9*
Udacha		V	3/25MS	4/80S	3/40MS	4/10S	4/50S	*Lr3a, Lr9, Lr20*
Polushko		III	4/60S	4/80S	4/70S	4/70S	4/80S	*Lr1, Lr3a, Lr10*
Baganskaya-93		II	4/80S	4/90S	4/50S	4/10S	4/50S	*Lr17a, Lr20*
Sirena	Krasnoyarskii krai / Krasnoyarskii NIISKH	V	2/15MR	2/10MR	3/40MS	2/5MR	2/15MR	*Lr3a*
Krasa-2		III	4/60S	4/60S	4/80S	4/70S	4/80S	*Lr3a, Lr10*
Krasnoyarskaya-90		I	4/80S	4/80S	4/70S	4/80S	4/90S	*Lr3a, Lr20*
Vesnyanka-8		III	4/90S	4/90S	4/70S	4/70S	4/90S	*Lr3a, Lr10, Lr20*
Albidum-73		V	4/60S	4/70S	4/70S	4/60S	4/80S	*Lr3a, Lr20*
Rybinskaya-127		II	4/60S	4/90S	4/80S	4/70S	4/90S	*Lr1*
Kazachka		V	4/80S	4/90S	4/80S	4/60S	4/70S	*Lr3a*
Angarida		III	4/70S	4/80S	4/70S	4/60S	4/60S	*Lr3a*
Mana-2		I	4/60S	4/80S	4/70S	4/60S	4/70S	*Lr3a*
Tuleevskaya	Kemerovskaya Oblast / Kemerovskii NIISKH	V	2/15MR	4/70S	4/80S	4/10S	4/10S	*Lr9*
Izida		V	4/80S	4/90S	4/90S	4/70S	4/90S	*Lr3a, Lr16*
Mariya		I	4/60S	4/80S	4/70S	4/15S	4/30S	*Lr3a, Lr10, Lr17a*
AN-34		V	3/20MS	3/20MS	3/40MS	3/5MS	3/20MS	*Lr1, Lr9, Lr10, Lr17a, Lr20*
Mariinka		I	4/60S	4/50S	3/60S	4/15S	4/30S	*Lr10, Lr16, Lr20*
Salimovka		III	4/60S	4/80S	4/80S	4/60S	4/60S	*Lr3a, Lr10, Lr16*
Kiiskaya		V	2/25MR	3/30MS	3/40MS	3/30MS	3/30MS	*Lr1, Lr3a, Lr9*
Nostalgiya		V	4/60S	4/80S	4/80S	4/40S	4/60S	*Lr3a, Lr10*
Aleshina		II	4/60S	4/90S	4/70S	4/15S	4/40S	*Lr3a, Lr10*
Darnitsa		III	4/70S	4/90S	4/70S	4/30S	4/40S	*Lr3a, Lr10*
Serebrina	Tyumenskaya Oblast / NIISKH Severnogo Zauralya	II	3/70MS	4/70S	4/50S	3/60MS	4/60S	*Lr3a, Lr20*
Rechka		I	4/60S	4/80S	4/70S	4/30S	4/50S	*Lr1, Lr3a, Lr17a*
Latona		IV	4/80S	4/80S	4/70S	4/20S	4/40S	*Lr3a, Lr17a*
Provintsiya		III	4/80S	4/90S	4/70S	4/30S	4/50S	*Lr1, Lr3a, Lr10*
Bel		III	4/70S	4/70S	4/70S	4/40S	4/50S	*Lr1, Lr3a, Lr10, Lr16*
Ustya		III	4/60S	4/70S	4/50S	4/15S	4/30S	*Lr3a, Lr10*
Chernyava-13		V	4/70S	4/80S	4/70S	4/60S	4/60S	*Lr1, Lr3a, Lr10*
Zlatozara		IV	4/60S	4/80S	4/70S	4/20S	4/40S	*Lr1, Lr3a*
Tyumenskaya-99		III	4/70S	4/80S	4/70S	4/30S	4/50S	*Lr10, Lr16*
Ikar		II	2/10MR	4/80S	4/60S	4/10S	4/30S	*Lr3a, Lr17a*
Skent-1		IV	4/70S	4/90S	4/70S	4/70S	4/70S	*Lr3a, Lr20*
Ilinskaya		IV	4/80S	4/80S	4/50S	4/60S	4/60S	*Lr3a, Lr10*
Turinskaya		I	3/20MS	4/40S	3/50MS	3/10MS	3/20MS	*Lr3a, Lr10, Lr17a*
Surenta-1		II	4/90S	4/90S	4/60S	4/50S	4/50S	*Lr3a, Lr17a*
Surenta-4		IV	4/60S	4/80S	4/50S	4/20S	4/40S	*Lr1, Lr20*
Surenta-5		IV	4/80S	4/90S	4/70S	4/70S	4/80S	*Lr3a*
Surenta-6		I	4/80S	4/90S	4/60S	4/30S	4/40S	*Lr3a*
Surenta-7		I	4/60S	4/90S	4/70S	4/60S	4/80S	*Lr3a, Lr10*
Dias-2	Omskaya Oblast / Sibirskii NIISKH	III	4/70S	4/90S	4/70S	4/15S	4/50S	*Lr3a, Lr10*
Irtyshanka-10		IV	4/70S	4/80S	4/70S	4/70S	4/70S	*Lr1*
Katyusha		IV	4/80S	4/90S	3/60MS	4/40S	3/40MS	*Lr3a, Lr34*
Tarskaya-6		IV	4/70S	4/80S	4/70S	4/70S	4/90S	*Lr3a, Lr10*
Sonata		V	2/30MR	4/20S	2/15MR	0/R	0/R	*Lr1, Lr3a*
Strada-Sibiri		I	4/80S	4/90S	4/90S	4/70S	4/80S	*Lr20, Lr34*
Otrada-Sibiri		I	2/10MR	0/R	2/10MR	0/R	0/R	*Lr3a, Lr10, Lr17a, Lr34*
Tertsiya		I	2/10MR	2/10MR	1/5MR	0/R	0/R	*Lr10*
Priirtyshskaya-86		III	4/10S	4/30S	3/40MS	4/10S	4/30S	*Lr3a, Lr10*
Rosinka-2		I	4/80S	4/90S	4/70S	4/40S	4/50S	*Lr3a, Lr10, Lr17a*
Omskaya-20		III	3/15MS	2/20MR	2/30MR	3/30MS	3/30MS	*Lr1, Lr3a, Lr20, Lr26*
Omskaya-23		I	2/10MR	3/50MS	2/40MR	2/10MR	2/20MR	*Lr3a, Lr10, Lr20*
Omskaya-24		I	4/70S	4/80S	4/60S	4/30S	4/50S	*Lr3a, Lr10, Lr20*
Omskaya-26		III	4/70S	4/80S	4/60S	4/30S	4/50S	*Lr3a, Lr10*
Omskaya-28		IV	4/70S	4/80S	4/70S	4/30S	4/50S	*Lr17a*
Omskaya-29		I	3/20MS	3/30MS	3/50MS	3/10MS	3/30MS	*Lr1, Lr3a, Lr10, Lr26, Lr34*
Omskaya-31		IV	4/70S	4/80S	4/70S	4/40S	4/50S	*Lr3a, Lr17a*
Omskaya-32		I	4/60S	4/80S	3/60MS	4/10S	4/30S	*Lr1, Lr3a*
Omskaya-33		IV	4/60S	4/80S	4/60S	4/60S	4/70S	*Lr1, Lr17a*
Omskaya-34		IV	3/40MS	3/40MS	3/40MS	3/20MS	3/20MS	*Lr1, Lr3a, Lr10, Lr17a*
Omskaya-36		IV	4/90S	4/90S	4/70S	4/70S	4/70S	*Lr1, Lr3a, Lr16*
Skala	Irkutskaya oblast / Tulunskaya GSS	III	4/90S	4/80S	4/70S	4/70S	4/70S	*Lr10*
Tulun-15		III	4/60S	4/70S	4/70S	4/70S	4/70S	*Lr10*

^a^ Genetic clustering is presented according to the results of the STRUCTURE program

^b^ Infection type was scored according to [27]. Severity ratings were scored as: *R* Resistant, *MR* Moderate resistant, *MS* Moderate susceptible, *S* susceptible; numeric character means % of leaf coverage by uredinia

**Table 2:** Analysis of variance of leaf rust resistance in spring wheat varieties

	DF	SS	MS	F value
Variety	99	270,454	2732	16.62***
Environments	4	48,143	12,036	73.23***
Field-1vsField-2	1	35,123	35,123	179.45***
Error	396	65,077	164	

*** Significant at *p* < 0.0001

**Fig. 1:**
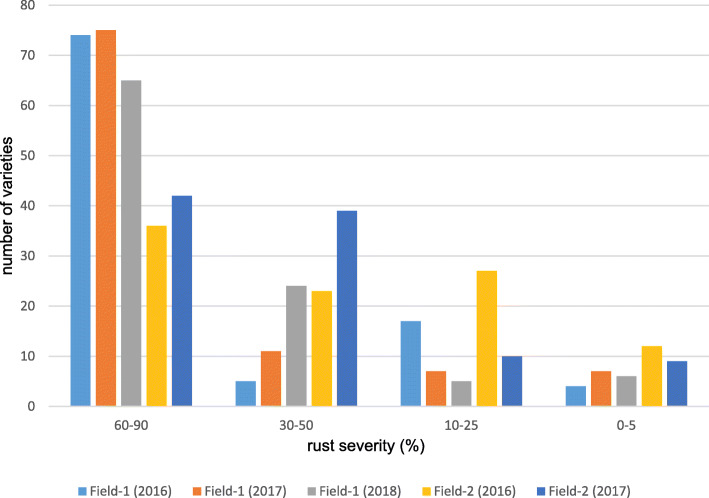
Diagram of distribution of spring wheat varieties by susceptibility to leaf rust on the experimental trials Field-1 and Field-2 in 2016–2018

Despite the same type of response to the pathogen, varieties cultivated under Field-2 conditions had a lower degree of leaf coverage with urediniospores compared to Field-1 (Table 1). Thus, in half of the samples, the degree of infection in Field-1 varied from 10 to 40%, while in Field-2 the same level of susceptibility was observed only in 21–33 varieties depending on the year of field evaluation (Fig. 1).

The number of highly and moderately-resistant varieties did not exceed 10% in different environmental conditions, with three varieties (Tulaikovskaya-zolotistaya, Tulaikovskaya-10, Samsar), created in Samarskii NIISKH, characterized as immune in all years of investigation. Based on the results of phytopathological assessments, varieties Tulaikovskaya-1, Volgouralskaya, Obskaya-14, Sonata, Sirena, Otrada-Sibiri, and Tertsiya were also noted, which showed low ITs in the conditions of Field-2.

### Population structure and association mapping

The population structure of wheat varieties was analyzed using 5950 SNP markers. The largest number of markers was used for genome B (2615 markers) compared to genomes A (2060) and D (736). According to the data obtained using the programs STRUCTURE and STRUCTURE HARVESTER, an assumption was made that the optimal number of subclusters is 5. Analysis of the composition of the subclusters did not reveal a clear separation of cultivars according to their origin from different breeding companies (Table 1). Thus, varieties of the selection of SiBNIIRS (Novosibirskaya oblast) are presented in all five subclusters. Varieties created in the breeding companies of the Altai, Krasnoyarsk, Kemerovo, Tyumen and Omsk regions are part of four out of the five subclusters. Varieties developed in Samarskii NIISKH, with the exception of the breeding line Kuibyshevskaya-2, are grouped in two subclusters (I and IV).

In addition, principal component analysis (PCA) performed with the help of the PAST program [28] was used to clarify genetic relationships. The results of PCA also did not reveal a genetically significant distribution of wheat collection into clear subpopulations (Additional file 2: Figure S1). The first two main components included 14.2 and 8.3% of genetic variation, respectively. The lack of a clear differentiation of cultivars can be explained by the fact that, since the 1970s of last century, varieties created in NIISH Yugo-Vostoka (Saratov) have become widespread. These varieties have now been widely involved in breeding in the Siberian and Ural regions [29].

Genotyping of wheat varieties showed no amplification for 118 SNP markers out of 13,007. After filtering, the number of markers used for associative mapping was 9406 (Additional file 3: Table S2). The number of markers mapped to different chromosomes of A, B or D genomes differed significantly, the smallest number was observed for chromosomes of the 4th homoeological group. Two variants of the MLM model were used for GWAS, one of which (MLM-1) included population structure and kinship (*Q* + *K*), the other - MLM-2– only kinship (*K*). The quantile-quantile plots (QQ), illustrating the correspondence between the observed and expected *p*-values for the two models, is presented in Additional file 4: Figure S2.

**Fig. 2:**
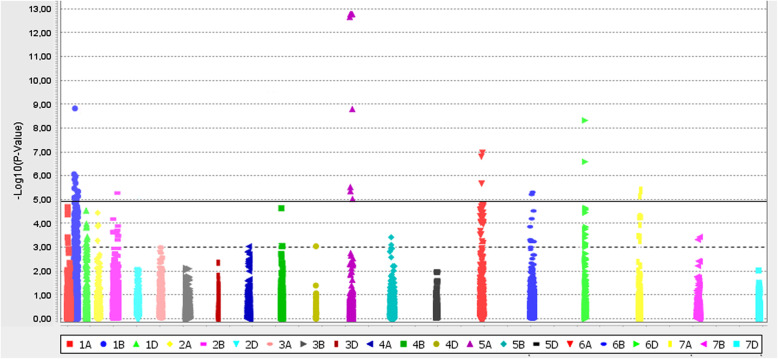
Manhattan plot illustrating the distribution of SNP associated with leaf rust resistance of wheat varieties. Markers located above the horizontal solid line are reliable at Bonferroni corrected *p*-value threshold (1.06 × 10^− 5^). Dashed line indicate *p*-value of 1 × 10^− 3^

Based on the results of GWAS, 20 SNP markers located on eight chromosomes (6D, 6A, 6B, 5A, 1B, 2A, 2B and 7A) were revealed, which showed reliable marker-trait association (Table 3, Fig. 2). The results presented in Table 3 indicate that MLM-1 and MLM-2 detected the same set of SNPs; the differences were only in the degree of reliability of MTAs, which were higher for the MLM-2 model.

**Table 3:** List and chromosomal localization of SNPs significantly associated with resistance to leaf rust

Marker	SNP ID	Chromosome^a^	Position, cM*	Allele^b^	*P* value MLM-1/MLM-2	R^2 c^	Effect
BS00063175_51	IWB9015	6DL	84.54	**G**/A	2.02E-05/4.81E-09	0.22–0.27	−3.4
BobWhite_c13435_700	IWB505	6DL	149.85		5.69E-08/9.48E-09	0.32–0.48	−3.90
BS00070856_51	IWB10505	6DL	153.07		2.83E-05/8.3E-06	0.18–0.29	−2.86
GENE-4153_101	IWB33802	6DL	82.14		7.37E-06/1.87E-06	0.20–0.27	−2.96
D_GB5Y7FA02G6KBX_382	IWB18070	6DL	83.75		9.71E-06/1.2E-06	0.22–0.27	−3.18
IAAV8633	IWB35508	6AS	3.43	**T**/C	1.80E-04/1.58E-07	0.11–0.20	−1.64
BS00037006_51	IWB8079	6АL	83.73	**C**/A	2.58E-05/2.31E-08	0.09–0.13	−1.52
wsnp_Ex_rep_c69526_68472787	IWA5615	5AS	35.94	**T/**G	6.05E-06/1.70E-13	0.24–0.27	−3.16
GENE-3321_201	IWB33331	5AS	36.72	**A**/C	6.05E-06/1.07E-13	0.24–0.27	−3.16
Kukri_c8835_112	IWB48151	5AS	36.58	**T**/C	6.05E-06/1.07E-13	0.20–0.27	−3.12
BS00094095_51	IWB11853	5AS	36.87	**T**/C	6.05E-06/1.45E-09	0.22–0.29	−2.48
tplb0023b14_704	IWB74145	1BL	70.07	**T**/G	2.15E-06/1.45E-09	0.17–0.26	−0.62
wsnp_Ra_c8484_14372815	IWA8082	1BL	69.76	**C**/T	2.15E-06/1.45E-09	0.17–0.26	−0.62
TA004947–0758	IWB65906	1BL	66.07	**C**/A	9.28E-06/2.54E-06	0.16–0.22	−0.64
BobWhite_c1456_615	IWB669	1BL	64.89	**C**/A	1.06E-05/2.04E-06	0.15–0.19	−0.58
RAC875_c8849_134	IWB60932	1BS	62.84	**C**/T	3.87E-05/9.78E-06	0.07–0.12	−0.31
wsnp_Ku_rep_c71900_71624324	IWA7504	1BS	–	**A**/G	1.15E-04/5.09E-06	0.08–0.12	−0.16
IAAV2452	IWB34561	1BS	62.63	**T**/C	3.49E-04/8.40E-06	0.04–0.09	−0.30
Excalibur_c18514_238	IWB23156	2AL	144.13	**G**/T	3.31E-05/5.68E-07	0.15–0.20	−0.87
Excalibur_c48404_59	IWB26954	2ВL	161.41	**T**/C	3.79E-05/8.25E-06	0.10–0.13	−0.54

^a^localization and marker position on the chromosome are indicated according to the consensus maps of wheat (*T. aestivum* L.) presented in [30] and Triticeae Toolbox database (https://triticeaetoolbox.org/)

^b^favorable alleles are highlighted in bold;

^c^R^2^ indicates phenotypic variation explained by the significant locus in different environments

Five significant markers with a high contribution to the trait manifestation in the range from − 2,86 to − 3,90 in three wheat varieties (Tulaikovskaya-zolotistaya, Tulaikovskaya-10, and Samsar) were revealed at chromosome 6D, among which four SNPs were null alleles (no amplification of the fragments in the genome of the varieties). Previously, we have shown for varieties Tulaikovskaya-5, Tulaikovskaya-10, and Tulaikovskaya-100 that the wheat chromosome 6D was replaced by the chromosome 6Ai = 2 from wheatgrass *Thinopyrum intermedium* [31]. Additionally, it was found that this wheatgrass chromosome carries at least one leaf rust resistance gene, *Lr6Ai = 2*, for which STS markers (Additional file 5: Table S3) were developed. Analysis of amplification of SNP markers, mapped to chromosome 6D, in varieties Tulaikovskaya-zolotistaya, Tulaikovskaya 10, and Samsar showed that 20% of the markers of the long arm are null alleles, which suggests the presence of chromosome substitution/translocation from *Th. intermedium*.

Two markers (IAAV8633 and BS00037006_51) that showed significant MTAs were located at the long and short arms of wheat chromosome 6A (Table 3). Favorable alleles of these markers with high reliability were detected only in varieties Tulaikovskaya-zolotistaya, Tulaikovskaya-10, and Samsar. Four informative SNPs with an effect on the phenotypic manifestation of the trait from − 2.48 to − 3.16 were identified at the 5AS chromosome. Favorable alleles were detected in the genomes of four varieties, Tulaikovskaya-zolotistaya, Tulaikovskaya-10, Samsar, and Volgouralskaya.

At the chromosome 1B, seven reliable MTAs were detected. Four of these SNPs were located at the long arm of the chromosome according to consensus maps. Favorable alleles of 1BL were found in the group of varieties including Sonata, Otrada-Sibiri, Tertsiya, Omskaya-23 developed in Sibirskii NIISKH, in Tulaikovskaya-1, Obskaya-14, and Sirena varieties created in the breeding centers Samarskii NIISKH, SibNIIRS and Krasnoyarskii NIISKH respectively. The favorable alleles for three SNPs located at the chromosome 1BS with minor effects from − 0.16 to – 0.31 were detected in varieties Kinelskaya-60, Omskaya-20 and Omskaya-29.

Significant association with resistance to leaf rust was shown for two markers mapped on chromosomes 2AL and 2BL. It should be noted that the favorable allele of the marker Excalibur_c18514_238 located at chromosome 2АL was revealed in the genome of Tulaikovskaya-zolotistaya, Tulaikovskaya 10, and Volgouralskaya varieties, whereas the favorable allele of the Excalibur_c48404_59 located at chromosome 2BL was found in Obskaya-14, Sonata, Tertsiya, Omskaya-23.

### Molecular marker analysis

The results of genotyping of wheat varieties with primers designed for the genes *Lr1*, *Lr3a*, *Lr9*, *Lr10*, *Lr16*, *Lr17a*, *Lr20*, *Lr21*, *Lr24*, *Lr25*, *Lr26*, *Lr28*, *Lr29*, *Lr34*, *Lr37*, and *Lr6Ai = 2* (Additional file 5: Table S3) are presented in Table 1. It transpired that most varieties contain genes *Lr1*, *Lr3a*, *Lr10*, *Lr17a*, and *Lr20* that are not currently effective. According to the marker analysis, these genes are postulated in the genome of 25, 75, 48, 24 and 21 varieties, respectively. There was no amplification of PCR fragments using primers specific to alien resistance genes *Lr24*, *Lr25*, *Lr28*, *Lr29* and *Lr37*. Seven varieties (Altaiskii-prostor, Altaiskaya-99, Aleksandrina, Udacha, Tuleevskaya, AN-34, and Kiiskaya) revealed a fragment of length 1110 bp using primer J13, developed for the gene *Lr9*. According to the published data obtained on the basis of molecular screening, varieties Altaiskaya-99, Udacha, Tuleevskaya, Aleksandrina, Sonata, and Tertsiya are carriers of *Lr9* [22, 23, 32]. However, no amplification of the fragment diagnostic to *Lr9* was found in Sonata and Tertsiya varieties in our study.

The PrCEN-2 and ω-sec-P1/P2 markers designed for the rye 1RS chromosome were used to identify the T1RS.1BL translocation containing the *Lr26* gene (Additional file 5: Table S3). Diagnostic fragments were amplified with DNA of three varieties: Kinelskaya-60, Omskaya-20, and Omskaya-29. In eight varieties, adult plant resistance gene *Lr34* was postulated based on the results obtained using the markers cssfr3 and csLV34 (Additional file 6: Figure S3). Identification of the *Lr6Ai = 2* gene from *Th. intermedium* was carried out using the STS marker *Xicg6Ai = 2*. PCR fragments indicating the presence of the wheatgrass genome are detected in the varieties Tulaikovskaya-zolotistaya, Tulaikovskaya-10, and Samsar (Additional file 6: Figure S4). In cv. Volgouralskaya, a 220 bp fragment was synthesized using the *Xwmc221* microsatellite marker, recommended for detecting the *Lr19* gene. The fact that the Volgouralskaya variety is a carrier of *Lr19* was shown using the *Xwmc221*, Gb, SCS265 and SCS253 markers in the investigations of other researchers [22, 23, 33].

## Discussion

This paper presents the results of an analysis of the diversity of Russian spring wheat varieties on genetic loci determining resistance to the leaf rust pathogen. Presence of resistance loci in the genome was postulated based on the comparison of data obtained using GWAS, amplification of PCR markers recommended for the detection of *Lr* genes, and phytopathological evaluation in different environments.

### Comparison of significant QTLs with known leaf rust resistance genes

The results of GWAS suggested the presence of the locus *Lr6Ai = 2* from *Th. intermedium* in the genomes of Tulaikovskaya-zolotistaya, Tulaikovskaya 10, and Samsar varieties (Table 1). PCR analysis using STS primers also confirmed the existence of *Lr6Ai = 2* in these cultivars (Additional file 6: Figure S4). Phytopathological evaluation suggests that the varieties demonstrated a type of reaction nearing full immunity in different environments, indicating a high efficiency of the gene *Lr6Ai = 2*.

In the Tulaikovskaya-zolotistaya, Tulaikovskaya-10, and Samsar varieties, reliable MTAs were detected for markers mapped to the 5AS, 6DL, 6AS and 6AL chromosomes (Table 3). Associations with SNPs of chromosome 5AS were also established for the Volgouralskaya variety. At present, the minor locus *QLr.pbi-5AS* is mapped on the short arm of chromosome 5A of bread wheat [34]. In addition, association mapping revealed a genomic region on chromosome 5A, which determines the resistance of durum wheat varieties to leaf rust [35]. Based on the location of QTLs, as well as their origin, the locus on 5AS found in this study cannot be attributed to any of the known genes. This suggests the presence of a previously unknown QTL for resistance to leaf rust in the genomes of Tulaikovskaya-zolotistaya, Tulaikovskaya-10, Samsar, and Volgouralskaya varieties.

Genes *Lr56*, *Lr62* and *Lr64* inherited from wheat relatives *Ae. sharonensis*, *Ae. neglecta*, and *T. dicoccoides* were transferred to chromosome 6A [4, 36, 37]. Associations of markers mapped to chromosome 6A may be explained by the presence of fragments of chromosome 6Ai from *Th. intermedium* both in chromosomes of the 6th homoeological group and in the other chromosomes of Tulaikovskaya-zolotistaya, Tulaikovskaya-10, and Samsar varieties. Such cases are described in the literature. For instance, for the gene *Lr38*, derived from *Th. intermedium*, several translocation lines with localization of the wheatgrass genome fragment 7Ai = 2 L in 3D, 6D, 1D, 2A chromosomes are known [38, 39]. For the *Lr9* gene inherited from *Ae. umbellulata* common wheat lines were obtained containing translocations in chromosomes 4BS, 2DL, 7BS [40]. However, to confirm this assumption additional cytological analysis is required.

Earlier, based on phytopathological tests confirmed by molecular analysis with Gb and SCS265 markers developed for the *Lr19* gene, it was suggested that cultivar Samsar is a carrier of this gene [21, 23, 41]. However, the results of PCR with primers for the gene *Lr6Ai = 2*, as well as the type of reaction to the pathogen, indicate that the resistance of the Samsar variety is determined by the gene *Lr6Ai = 2* (Additional file 6: Figure S4). Moreover, the use of the primer wmc221 did not reveal amplification of the fragment diagnostic for *Lr19*.

Three varieties (Kinelskaya-60, Omskaya-20, and Omskaya-29) are carriers of the gene *Lr26*, introduced as part of the wheat-rye translocation. This is evidenced by both GWAS results which indicated three reliable MTAs in chromosome 1BS, and molecular screening by markers PrCEN-2 and ω-sec-P1/P2. Pedigree analysis of these varieties shows that the cv. Caucasus – the source of *Lr26* gene (http://wheatpedigree.net/) took part in their creation. Additionally, it was shown that SNP marker TG0025, developed for 1RS/1BL translocation by TraitGenetics GmbH (personal communication), is detected only in these three varieties (*p* < 1.04E-05). For the Kinelskaya-60, Omskaya-20, and Omskaya-29 - carriers of the *Lr26* gene, the type of reaction is moderately susceptible (IT = 3/15-50MS), indicating a decrease in the efficacy of the gene (Table 1).

The presence of the *Lr9* gene was postulated in Altaiskii-prostor, Altaiskaya-99, Aleksandrina, Udacha, Tuleevskaya, AN-34, and Kiiskaya varieties by PCR results with the J13 marker. However, GWAS did not identify reliable MTAs (*p* < 0.001) specific for chromosomal localization of the gene *Lr9* inherited from *Ae. umbellulata* as part of the T6BS.6BL-6 U#1 L. Phytopathological screening indicates that all varieties showed susceptible response to the pathogen, indicating a loss of effectiveness of this gene to the native population of leaf rust.

Analysis of the GWAS results revealed that seven varieties (Sonata, Otrada-Sibiri, Tertsiya, Omskaya-23, Obskaya-14, Tulaikovskaya-1, and Sirena) are combined into a group characterized by a set of associated SNPs mapped to the long arm of chromosome 1B. According to phytopathological testing presented by Sing et al. [21], the gene *Lr23* inherited from *T. durum* was postulated in the genome of Tulaikovskaya-1. Taking into account the loss of efficacy of the *Lr23* and *Lr9* genes to *P. triticina* populations in Western Siberia [3, 32, 42], low susceptibility to leaf rust of Sonata, Otrada-Sibiri, Tertsiya, Omskaya-23, Obskaya-14, Tulaikovskaya-1, and Sirena may indicate the presence of an unknown locus in chromosome 1B, which provides a resistance response.

### Effectiveness of leaf rust resistance genes in Western Siberia

The obtained results indicate the absence of effective resistance genes to *Puccinia triticina* in most Russian spring wheat varieties. Only 15 varieties out of 100 are characterized by high or moderate resistance to the pathogen, of which three varieties - carrying the gene *Lr6Ai = 2* demonstrated an “immune” reaction type. The *Lr19* gene has retained its effectiveness, as evidenced by both the infection type of the cv. Volgouralskaya and the data obtained earlier using the RL6040 isogenic Thatcher line [3]. It can be assumed that the West Siberian population of *P. triticina* does not contain races that are virulent to *Lr19*; however, this requires additional study of the virulence of the local population. Nevertheless, it is clear that the phytopathological situation in the region is fundamentally different from other regions of Russia, where the protective effect of the *Lr19* gene is overcome. Currently, virulence to *Lr19* is registered in many Russian regions where varieties with this gene are cultivated [42–44].

Until recently, when creating varieties of spring wheat adapted for Western Siberia, hybridization schemes included mainly sources of genes *Lr9* and *Lr19* that have long maintained their effectiveness in many regions of the world [45–47]. There is evidence, based mainly on the data of hybridological analysis, that a number of varieties of local selection contain the gene *LrTr* = *Lr9* [48, 49]. However, intensive use of the same type of material with the *Lr9* gene led to the appearance of isolates virulent to this gene, and, accordingly, its loss of efficacy. This is confirmed by the results presented in this study, which indicate that the varieties carrying *Lr9* are characterized by a susceptible response to the pathogen.

A significant decrease in the effectiveness of the juvenile gene *Lr26* and the adult plant resistance gene *Lr34* and the appearance of virulent races was recorded in the Tyumen, Omsk and Novosibirsk regions, which are part of the Ural and West Siberian regions [42, 50, 51]. In our study, marker analysis led us to postulate the presence of *Lr34* in eight wheat varieties (Kuibyshevskaya-2, Altaiskaya-92, Altaiskaya-325, Ustya, Katyusha, Strada-Sibiri, Otrada-Sibiri, and Omskaya-29). However, for six of them, excluding Otrada-Sibiri and Omskaya-29, which contain genes *Lr26* and an unidentified gene in chromosome 1BL, high susceptibility to the pathogen was noted (IT = 4/15-90S; Table 1).

Monitoring of virulence of the West Siberian population conducted in 2007–2017, showed a high susceptibility for wheat samples containing leaf rust resistance genes *Lr1*, *Lr2a*, *Lr2b*, *Lr2c*, *Lr2d*, *Lr3a*, *Lr3bg*, *Lr3ka*, *Lr9*, *Lr10*, *Lr11*, *Lr14a*, *Lr15*, *Lr16*, *Lr17a*, *Lr20*, *Lr22a*, *Lr23*, *Lr26*, *Lr30*, *Lr32*, *Lr33*, *Lr34*, *Lr44* [3, 32]. It is important to note that there was a difference in infectious backgrounds (subpopulations of *P. triticina*) from Field-1 and Field-2, which consisted not so much in the composition of virulence genes, but in the degree of aggressiveness of the infection in the Field-1 microclimate. Immunity to the field population of the leaf rust was preserved by carriers of the *Lr19*, *Lr24*, *Lr28*, *Lr35*, *Lr45*, *Lr47*, *Lr50*, *Lr52* (*LrW*) genes. Our results suggest that the local population either does not contain the corresponding virulence genes or the resistance genes listed are able to effectively protect genotypes in the region.

## Conclusions

A comprehensive assessment carried out using genome-wide association study, molecular marker analysis and phytopathological evaluation, suggests that most of the studied Russian spring wheat varieties do not contain genes that provide effective resistance to *P. triticina*. The results indicate that the protective effects of the genes *Lr9* and *Lr26* have been overcome, whereas genes *Lr6Ai = 2* and *Lr19* retain their effectiveness. Based on the localization and origin of known leaf rust loci, QTLs on chromosomes 5AS and 1BL cannot be attributed to any of the known resistance genes. The SNPs, which showed reliable MTAs in this study, can be used for development of new markers for identification of leaf rust resistance loci.

## Methods

### Plant material and field experiments

The plant material included 100 varieties and breeding lines of spring bread wheat, created by eight breeding companies of the Russian Federation; the list includes both old-fashioned varieties and varieties of modern selection. The list of varieties is presented in Table 1 and Additional file 7: Table S4, more detailed pedigree information can be found in the GRIS Internet resources (Genetic Resources Information System for Wheat and Triticale, http://wheatpedigree.net) and in the database of the bioresource collections of the IC&G SB RAS (http://ckp.icgen.ru/plants/fond). Seeds were obtained from the National Genebank of Russian Federation (VIR, Federal Research Center N.I. Vavilov All-Russian Institute of Plant Genetic Resources, St. Petersburg; http://db.vir.nw.ru/virdb/maindb), were maintained and multiplied in The Federal Research Center Institute of Cytology and Genetics SB RAS (IC&G SB RAS, Novosibirsk).

Wheat varieties were grown in 2016–2017 on two experimental fields of IC&G, located in the Novosibirsk region and further designated as Field-1 (54.9191° N, 82.9903° E) and Field-2 (54.8475° N, 83.1095° E). In 2018, phytopathological screening for leaf rust susceptibility was conducted only on Field-1. Experimental plots are affected differently by wind. The open area of Field-1 provides the best assessment of the natural infection. The area of Field-2 is surrounded by forest and is not exposed to airborne spores of pathogen well. However here there is always good humidity, due to the duration and intensity of precipitation, stable dew and the period of leaf moistening, which makes it possible to evaluate the disease even in years unfavorable for the development of rust.

Varieties were sown in a randomized block design with two replications at each of two experimental fields. The seeds were planted in rows of 1 m long, the distance between rows was 25 cm and the distance between plants within a row was 5 cm. Field response to uncontrolled, natural infection of leaf rust was estimated twice per season at booting and early milk stages. Type of reaction and severity ratings were determined using the 0–4-point scale of Mains and Jackson [27] and a modified Cobb scale [52].

### Genotyping and DNA marker analysis

Genomic DNA was isolated from 5 to 7-day-old seedlings as described in [53]. For SNP genotyping, DNA was purified on micro columns from the “Bio-Silica” company according to the manufacturer’s instructions. Genotyping was carried out with the help of the Illumina Infinium 15 K array of TraitGenetics GmbH (www.traitgenetics.de), which included 13,007 SNP markers mapped in the wheat genome [30].

For postulation of the presence of *Lr* resistance genes, wheat genotypes were analyzed using PCR markers developed for genes *Lr1*, *Lr3a*, *Lr9*, *Lr10*, *Lr16*, *Lr17a*, *Lr20*, *Lr21*, *Lr24*, *Lr25*, *Lr26*, *Lr28*, *Lr29*, *Lr34*, *Lr37,* and *Lr6Ai =2*. Thatcher near isogenic lines and wheat cultivars containing *Lr* genes were used as controls. PCR was performed according to published protocols in a 20 μl reaction mixture containing 50 ng of DNA. The list of used markers, primer sequences and references are presented in Additional file 5: Table S3. PCR products were separated in a 1.5% agarose gel, stained with ethidium bromide and visualized on the Gel Doc digital gel documentation system (Applied Biosystems).

### Data analysis

Analysis of variance (ANOVA) of the data on leaf rust resistance in different environments was performed using the program STATISTICA v. 10 (www.statsoft.ru). The population structure (*Q*-matrix) was estimated using a Bayesian algorithm implemented in the program STRUCTURE 2.3.4 [54]. *Q*-matrix was calculated based on the results of genotyping with 5950 SNP markers. The number of suspected subclusters ranged from 1 to 10. The simulation was performed using the admixture model; the number of runs was five with a burn-in length of 20,000 and Markov chain iterations of 50,000. The most likely number of clusters is calculated from Delta *K* (Δ*K*) statistics [55] using the web-based program Structure Harvester [56]. Principal component analysis (PCA) implemented in the PAST v. 3.15 program was used to group the accessions based on genetic similarity [28]. Kinship (*K*) matrix was calculated using the program TASSEL V. 5.2.24 [57]. A complete set of SNP markers was used to calculate the K-matrix, with the exception of markers that showed missing data for all analyzed samples.

Marker-trait associations (MTAs) were determined on the basis of mixed linear model (MLM) with kinship matrix (*K*) and population structure (*Q*) as covariate using the program TASSEL v. 5.2.24. SNP markers with MAF (minor allele frequency) less than 5% and missing data > 20% were not included in the analysis. After filtering, the number of markers was 9406. Two criteria were used to identify reliable MTAs: 1) Bonferroni multiple correction with α = 0.1, which corresponded to marker-wise probability *p* < 1.06 × 10^–^5; 2) a false discovery rate (FDR) of *p* < 0.001 as the threshold to identify markers associated with resistance, taking into account only markers that showed associations in at least two environments. The proposed genetic location of QTLs associated with resistance was determined using consensus maps of hexaploid wheat chromosomes presented in Wang et al. [30].

## Supplementary information

**Additional file 1: Table S1.** Meteorological conditions for the growing seasons of 2016, 2017 and 2018 (Novosibirskaya oblast, weather station 54.54° N, 82.57° E).

**Additional file 2: Figure S1.** A scatter plot of PCA of the spring wheat varieties obtained on the base of SNP genotyping.

**Additional file 3: Table S2.** Number of SNP markers with localizations in the A, B and D genomes used for genotyping wheat varieties.

**Additional file 4: Figure S2.** Quantile – quantile plots demonstrating the ratios of expected to observed log10 (P) values.

**Additional file 5: Table S3.** List of primers used for postulation of *Lr* genes in spring wheat varieties.

**Additional file 6: Figure S3.** PCR profile of primer cssfr3 for *Lr34* gene with DNA wheat varieties Kuibishevskaya-2, Altaiskaya-92, Altaiskaya-325, Ustya, Katyusha, Otrada-Sibiri and isogenic Thatcher line RL6058. **Figure S4.** Electrophoretic image of PCR fragments obtained by amplification of DNA of wheat varieties with primer *Xicg6Ai = 2* developed for the gene *Lr6Ai = 2*.

**Additional file 7: Table S4.** Wheat accessions used in the genome-wide association study (GWAS) for leaf rust severities.

## Abbreviations

ANOVA: Analysis of variance;
GWAS: Genome-wide association study;
IT: Infection type
LD: Linkage disequilibrium
Lr: Leaf rust;
MAF: Minor allele frequency;
MAS: Marker-assisted selection;
MLM: Mixed linear model;
MTA: Marker-trait association;
QQ: Quantile-quantile;
QTL: Quantitative trait loci;
SNP: Single nucleotide polymorphism
SR: Severity ratings
