# Supplementary material for: Genome-wide association study of leaf rust resistance in Russian spring wheat varieties
Source: BMC Plant Biol. 2020 Oct 14;20(Suppl 1):135. doi: 10.1186/s12870-020-02333-3 (PMC7557001; doi:10.1186/s12870-020-02333-3)
Supplement: Supplementary file 1 — Additional file 1: Table S1. Meteorological conditions for the growing seasons of 2016, 2017 and 2018 (Novosibirskaya oblast, weather station 54.54° N, 82.57° E). [file 12870_2020_2333_MOESM1_ESM.docx]

**Table S1.** Meteorological conditions for the growing seasons of 2016, 2017 and 2018 (Novosibirskaya oblast, weather station 54.54° N, 82.57° E).

| Month | Decade | Temperature (^o^C) | | | | Precipitation, mm | | | |
| --- | --- | --- | --- | --- | --- | --- | --- | --- | --- |
|  |  | 2016 | 2017 | 2018 | Long-term average indicator | 2016 | 2017 | 2018 | Long-term average indicator |
| May | I | 6.5 | 8.7 | 5.0 | 8.7 | 7.0 | 12.5 | 28.2 | 14.0 |
|  | II | 8.7 | 12.3 | 6.0 | 10.8 | 11.1 | 13.7 | 25.8 | 13.0 |
|  | III | 15.8 | 16.4 | 9.6 | 12.9 | 13.5 | 7.7 | 26.2 | 10.0 |
| June | I | 17.5 | 16.5 | 17.9 | 15.0 | 0.0 | 25.5 | 74.1 | 18.0 |
|  | II | 20.5 | 20.0 | 18.3 | 17.3 | 28.1 | 10.5 | 39.6 | 16.0 |
|  | III | 21.1 | 21.5 | 21.0 | 18.6 | 9.6 | 36.0 | 16.5 | 21.0 |
| July | I | 19.9 | 17.8 | 18.0 | 19.3 | 46.7 | 49.8 | 9.9 | 16.0 |
|  | II | 21.0 | 17.8 | 21.5 | 19.7 | 15.6 | 16.9 | 0.3 | 19.0 |
|  | III | 19.8 | 19.8 | 16.2 | 19.2 | 14.4 | 32.8 | 54.4 | 26.0 |
| August | I | 18.2 | 20.1 | 15.7 | 17.6 | 12,2 | 20.0 | 3.3 | 26.0 |
|  | II | 18.0 | 14.0 | 18.5 | 16.9 | 0.0 | 36.3 | 2.2 | 18.0 |
|  | III | 15.9 | 16.5 | 15.6 | 14.4 | 7.8 | 9.3 | 27.8 | 23.0 |
