# Supplementary material for: Genome-wide association study of leaf rust resistance in Russian spring wheat varieties
Source: BMC Plant Biol. 2020 Oct 14;20(Suppl 1):135. doi: 10.1186/s12870-020-02333-3 (PMC7557001; doi:10.1186/s12870-020-02333-3)
Supplement: Supplementary file 2 — Additional file 2: Figure S1. A scatter plot of PCA of the spring wheat varieties obtained on the base of SNP genotyping. [file 12870_2020_2333_MOESM2_ESM.docx]

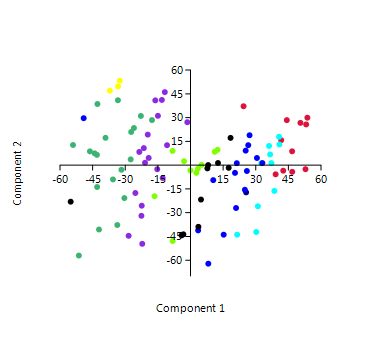


**Figure S1**. A scatter plot of PCA of the spring wheat varieties obtained on the base of SNP genotyping. Varieties belonging to the organization-originator are marked with the following color: red - Samarskii NIISKH; blue – Altaiskii NIIZIS; dark blue – SibNIIRS; black – Krasnoyarskii NIISKH; green – Kemerovskii NIISKH; violet – NIISKH Severnogo Zauralya; dark green – Sibirskii NIISKH; yellow – NIISKH Yugo-Vostoka.
