# Supplementary material for: Genome-wide association study of leaf rust resistance in Russian spring wheat varieties
Source: BMC Plant Biol. 2020 Oct 14;20(Suppl 1):135. doi: 10.1186/s12870-020-02333-3 (PMC7557001; doi:10.1186/s12870-020-02333-3)
Supplement: Supplementary file 3 — Additional file 3: Table S2. Number of SNP markers with localizations in the A, B and D genomes used for genotyping wheat varieties. [file 12870_2020_2333_MOESM3_ESM.docx]

**Table S2.** Number of SNP markers with localizations in the A, B and D genomes used for genotyping wheat varieties.

| Genome | Chromosome | | | | | | | Total |
| --- | --- | --- | --- | --- | --- | --- | --- | --- |
|  | 1 | 2 | 3 | 4 | 5 | 6 | 7 |  |
| A | 602 | 448 | 502 | 441 | 502 | 574 | 582 | 3651 |
| B | 639 | 853 | 655 | 230 | 790 | 706 | 506 | 4379 |
| D | 251 | 390 | 131 | 59 | 193 | 195 | 157 | 1376 |
