# Supplementary material for: Genome-wide association study of leaf rust resistance in Russian spring wheat varieties
Source: BMC Plant Biol. 2020 Oct 14;20(Suppl 1):135. doi: 10.1186/s12870-020-02333-3 (PMC7557001; doi:10.1186/s12870-020-02333-3)
Supplement: Supplementary file 4 — Additional file 4: Figure S2. Quantile – quantile plots demonstrating the ratios of expected to observed log10 (P) values. [file 12870_2020_2333_MOESM4_ESM.docx]

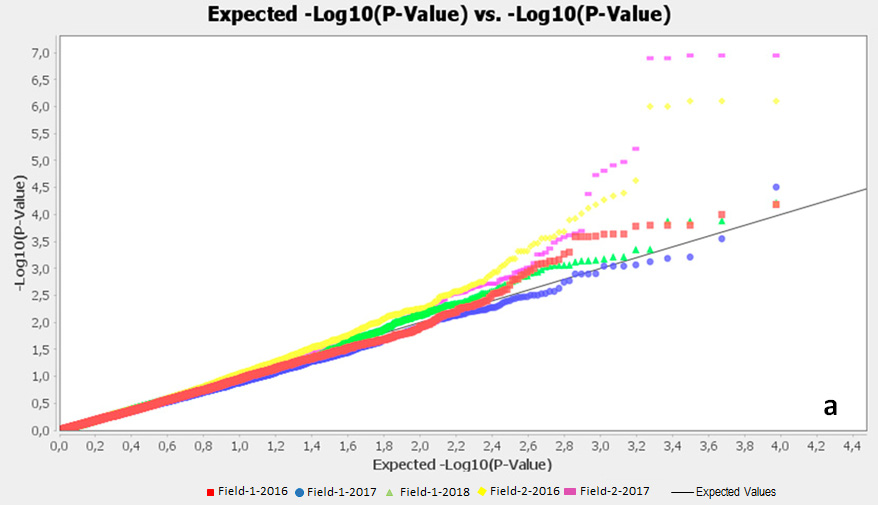

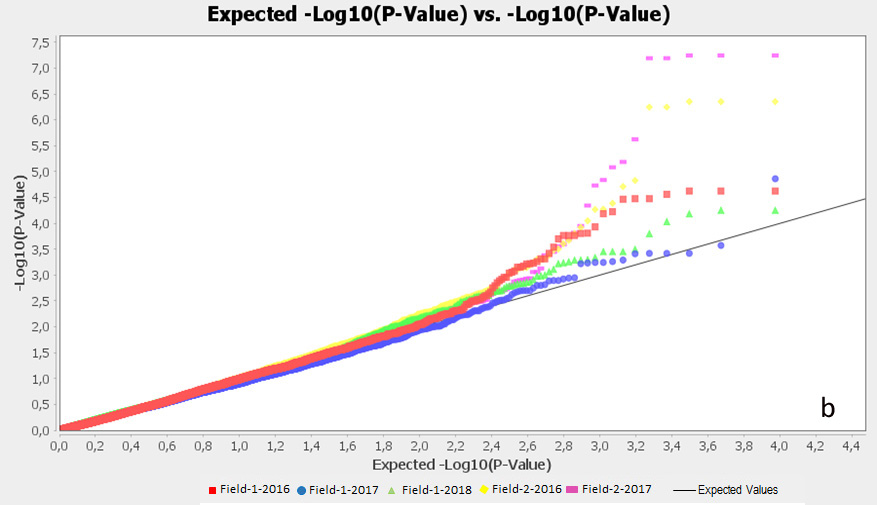


**Figure S2.** Quantile – quantile plots demonstrating the ratios of expected to observed log10 (P) values: a) model MLM-1 (*Q* + *K*); b) Model MLM-2 (*K*)
