# Supplementary material for: Genome-wide association study of leaf rust resistance in Russian spring wheat varieties
Source: BMC Plant Biol. 2020 Oct 14;20(Suppl 1):135. doi: 10.1186/s12870-020-02333-3 (PMC7557001; doi:10.1186/s12870-020-02333-3)
Supplement: Supplementary file 5 — Additional file 5: Table S3. List of primers used for postulation of Lr genes in spring wheat varieties. [file 12870_2020_2333_MOESM5_ESM.docx]

**Table S3.** List of primers used for postulation of *Lr* genes in spring wheat varieties

| *Lr* gene | Marker | Primer structure (5’ → 3’) | Reference |
| --- | --- | --- | --- |
| *Lr1* | WR003 | F-GGGACAGAGACCTTGGTGGA  R-GACGATGATGATTTGCTGCTGG | [1] |
| *Lr3a* | *Xmwg798* | F-GGCTGTCTACATCTTCTGCA  R CAAGTGTTGAGAAGGAGAGT | [2] |
| *Lr9* | J13 | F- CCACAСTACCCCAAAGAGACG  R- TCCTTTTATTCCGCACGCCGG | [3] |
| *Lr10* | F1.2245/  Lr10-6/r2 | F-GTG TAA TGC ATG CAG GTT CC  R-AGG TGT GAG TGA GTT ATG TT | [4] |
| *Lr16* | pwm6  pwm16 | F-AGTGCAGAGCAGAGGCAAAC  R-TGCCATGGGCTCTACCATAC  F-CCTACCTAACCAGCGTGCTC  R-CTGTGTAGACGGCGATACGA | [5] |
| *Lr17a* | *Xbarc124* | F-TGCACCCCTTCCAAATCT  R-TGCGAGTCGTGTGGTTGT | [6] |
| *Lr19* | *Xwmc221* | F-ACGATAATGCAGCGGGGAAT  R-GCTGGGATCAAGGGATCAAT | CIMMYT, personal commun. |
| *Lr20* | STS638 | L-GCGGTGACTACACAGCGATGAAGCAATGAAA  R-GCGGTGACTAGTCCAGTTGGTTGATGGAAT | [7] |
| *Lr21* | D14 | L- CGCTTTTACCGAGATTGGTC  R- CCAAAGAGCATCCATGGTGT | [8] |
| *Lr24* | Sr24#12  SCS73 | F-CACCCGTGACATGCTCGTA  R-AACAGGAAATGAGCAACGATGT  F-TCGTCCAGATCAGAATGTG  R-CTCGTCGATTAGCAGTGAG | [9]  [10] |
| *Lr25* | Lr25 | F20-CCACCCAGAGTATACCAGAG  R19- CCACCCAGAGCTCATAGA | [11] |
| *Lr26* | PrCEN-2  ω-sec-P1/P2 | F-AATGATCTTCCACGACGACG  R-CCTCGTTGGGAAATGGTGCA  F-ACCTTCCTCATCTTTGTCCT  R-CCGATGCCTATACCACTACT | [12] |
| *Lr28* | sts421 | F-ACAAGGTAAGTCTCCAACCA  R- AGTCGACCGAGATTTTAACC | [13] |
| *Lr29* | Lr29 | F24-GTGACCTCAGGCAATGCACACAGT  R24-GTGACCTCAGAACCGATGTCCATC | [11] |
| *Lr34* | cssfr3  csLV34 | F-TTGATGAAACCAGTTTTTTTTCTA  R-GCCATTTAACATAATCATGATGGA  F-GTTGGTTAAGACTGGTGATGG  R- TGCTTGCTATTGCTGAATAGT | [14] |
| *Lr37* | VENTRUP-LN2 | VENTRUP – AGGGCTACTGACCAAGGCTC  LN2 - TGCAGCTACAGCAGTATGTACACAAAA | [15] |
| *Lr6Ai#2* | *Xicg6Ai=2* | F- GATGTCGAGGAGCATTTTC´  R- GTGGTAGATTACTAGAGTTCAAGTG | [16] |

1. Qui JW, Schürch AC, Yahiaoui N, Dong LL, Fan HJ, Zhang ZJ, Keller B, Ling HQ. Physical mapping and identification of a candidate for leaf rust resistance gene *Lr1* of wheat Theor Appl Genet. 2007;115:159-68.
2. Sacco F, Suárez EY, Naranjo T. Mapping of the leaf rust resistance gene *Lr3* on chromosome 6B of Sinvalocho MA wheat. Genome. 1998;41:686-90.
3. Schachermayr G, Sieder H, Gale MD, Winzeller H, Winzeller M, Keller B. Identification and localization of molecular markers linked to the *Lr9* leaf rust resistance gene of wheat. Theor Appl Genet. 1994;88:110-15.
4. Schachermayr G, Feuillet C, Keller B. Molecular markers for the detection of the wheat leaf rust resistance gene *Lr10* in diverse genetic backgrounds. Mol Breed. 1997;3:65-74.
5. Kassa MT, You FM, Hiebert CW, Pozniak CJ, Fobert PR, Sharpe AG, Menzies JG, Humphreys DG, Rezac HR, Fellers JP, McCallum BD, McCartney CA. Highly predictive SNP markers for efficient selection of the wheat leaf rust resistance gene *Lr16*. BMC Plant Biol. 2017;17:45
6. Zhang JX, Singh RP, Kolmer JA, Huerta-Espino J, Jin Y, Anderson JA. Inheritance of leaf rust resistance in the CIMMYT wheat Weebill. Crop Sci. 2007;48:1037-1047.
7. Neu C, Stein N, Keller B. Genetic mapping of the *Lr20*-*Pm1* resistance locus reveals suppressed recombination on chromosome arm 7AL in hexaploid wheat. Genome. 2002;45:737-44.
8. Talbert LE; Blake NK; Chee PW; Blake TK; Magyar GM. **Evaluation of "sequence-tagged-site" PCR products as molecular markers in wheat**. Theor Appl Genet. 1994;87:789-94.
9. Mago R, Bariana HS, Dundas IS, Spielmeyer W, Lawrence GJ, Pryor AJ, Ellis JG. Development or PCR markers for the selection of wheat stem rust resistance gene *Sr24* and *Sr26* in diverse wheat germplasm. Theor Appl Genet. 2005;111:496-504.
10. Prabhu KV, Gupta SK, Charpe A, Koul S. SCAR marker tagged to the alien leaf rust resistance gene *Lr19* uniquely marking the *Agropyron elongatum*-derived gene *Lr24* in wheat: a revision. Plant Breed. 2004;123:417-20.
11. Procunier JD, Townley-Smith TF, Fox S, Prashar S, Gray M, Kim WK, Czarnecki E, Dyck PL. **PCR-based RAPD/DGGE markers linked to leaf rust resistance genes *Lr29* and *Lr25* in wheat (*Triticum aestivum* L.)**. J Genet Breed. 1995;49:87-92.
12. Ren T, Tang Z, Fu S, Yan B, Tan F, Ren Z and Li Z. Molecular cytogenetic characterization of novel wheat-rye T1RS.1BL translocation lines with high resistance to diseases and great agronomic traits. Front Plant Sci. 2017;8:799.
13. Cherukuri DP, Gupta SK, Charpe A, Koul S, Prabhu KV, Singh RB, Haq QMR. Molecular mapping of *Aegilops speltoides* derived leaf rust resistance gene *Lr28* in wheat. Euphytica. 2005;143:19-26.
14. Lagudah ES, Krattinger SG, Herrera-Foessel S, Singh RP, Huerta- Espino J, Spielmeyer W, Brown-Guedira G, Selter LL, Keller B. Gene-specific markers for the wheat gene Lr34/Yr18/ Pm38 which confers resistance to multiple fungal pathogens. Theor Appl Genet. 2009;119:889-98.
15. Helguera M, Khan IA, Kolmer J, Lijavetzky D, Zhong-Qi L, Dubcovsky J. PCR assays for the *Lr37-Yr17-Sr38* cluster of rust resistance genes and their use to develop isogenic hard red spring wheat lines. Crop Sci. 2003;43:1839-47.
16. Skolotneva ES, Leonova IN, Bukatich EYu, Salina EA. Methodical approaches to identification of effective wheat genes providing broad-spectrum resistance against fungal diseases. Vavilovskii Zhurnal Genetiki i Selektsii = Vavilov J Genet Breed. 2017;21:862-9.
