# Supplementary material for: Genome-wide association study of leaf rust resistance in Russian spring wheat varieties
Source: BMC Plant Biol. 2020 Oct 14;20(Suppl 1):135. doi: 10.1186/s12870-020-02333-3 (PMC7557001; doi:10.1186/s12870-020-02333-3)
Supplement: Supplementary file 6 — Additional file 6: Figure S3. PCR profile of primer cssfr3 for Lr34 gene with DNA wheat varieties Kuibishevskaya-2, Altaiskaya-92, Altaiskaya-325, Ustya, Katyusha, Otrada-Sibiri and isogenic Thatcher line RL6058. Figure S4. Electrophoretic image of PCR fragments obtained by amplification of DNA of wheat varieties with primer Xicg6Ai = 2 developed for the gene Lr6Ai = 2. [file 12870_2020_2333_MOESM6_ESM.docx]

**
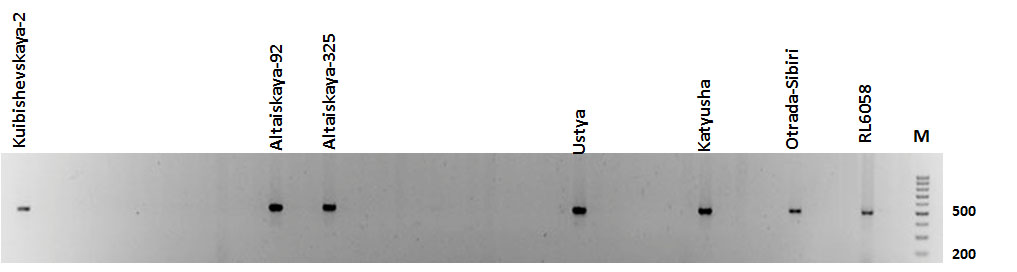
**

**Figure S3.** PCR profile of primer cssfr3 for *Lr34* gene with DNA of wheat varieties Kuibishevskaya-2, Altaiskaya-92, Altaiskaya-325, Ustya, Katyusha, Otrada-Sibiri and isogenic Thatcher line RL6058. Lane M – DNA ladder.


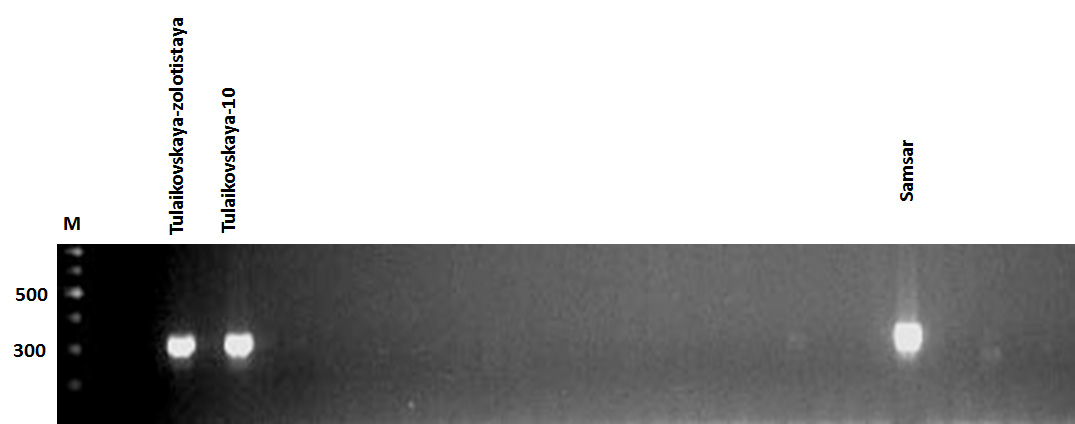


**Figure S4.** Electrophoretic image of PCR fragments obtained by amplification of DNA of wheat varieties with primer *Xicg6Ai=2* developed for the gene *Lr6Ai=2*. Lane M – DNA ladder.
